# Finite Element Modeling and Vibration Control of Plates with Active Constrained Layer Damping Treatment

**DOI:** 10.3390/ma16041652

**Published:** 2023-02-16

**Authors:** Zhicheng Huang, Huanyou Peng, Xingguo Wang, Fulei Chu

**Affiliations:** 1 College of Mechanical and Electrical Engineering, Jingdezhen Ceramic University, Jingdezhen 333001, China; 2 Department of Mechanical Engineering, Tsinghua University, Beijing 100084, China

**Keywords:** active constrained layer damping, finite element modeling, model order reduction, active control, viscoelastic material

## Abstract

An enhanced lightness and thinness is the inevitable trend of modern industrial production, which will also lead to prominent low-frequency vibration problems in the associated structure. To solve the vibration problem of thin plate structures in various engineering fields, the active constrained layer damping (ACLD) thin plate structure is taken as the research object to study vibration control. Based on the FEM method, energy method, and Hamilton principle, the dynamic model of an ACLD thin plate structure is derived, in which the Golla–Hughes–McTavish (GHM) model is used to characterize the damping characteristics of the viscoelastic layer, and the equivalent Rayleigh damping is used to characterize the damping characteristics of the base layer. The order of the model is reduced based on the high-precision physical condensation method and balance reduction method, and the model has good controllability and observability. An LQR controller is designed to actively control the ACLD sheet, and the controller parameters and piezoelectric sheet parameters are optimized. The results show that the finite element model established in this paper is accurate under different boundary conditions, and the model can still accurately and reliably describe the dynamic characteristics of the original system in the time and frequency domain after using the joint reduction method. Under different excitation and boundary conditions, LQR control can effectively suppress structural vibration. Considering the performance and cost balance, the most suitable control parameter for the system is: Q-matrix coefficient is between 1 × 10^4^ and 1 × 10^5^, the R-matrix coefficient is between 1 and 10, and the thickness of the piezoelectric plate is 0.5 mm.

## 1. Introduction

**Figure 1:**
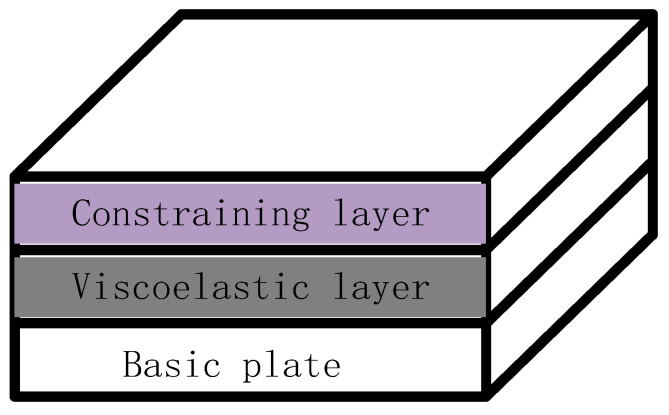
The structure of PCLD treatment.

**Figure 2:**
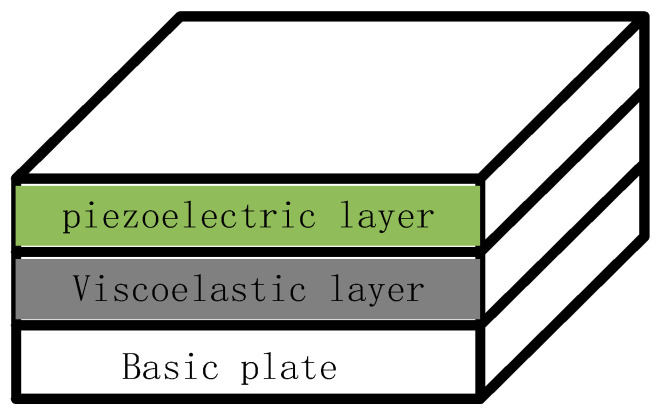
The structure of ACLD treatment.

With the rapid development of modern industry and the progress of engineering technology, the number of industrial products used in daily life has gradually increased, such as vehicles, aircraft, and ships. The negative impact of vibrations should be emphatically considered in the design of such products. System structure vibration has become a hot issue in today’s society [1,2]. The commonly used control methods are divided into three main categories: passive control, active control, and integrated active and passive control [3,4,5]. A common passive control structure is passive constrained layer damping (PCLD) [6]. As shown in Figure 1, when a PCLD structure is excited by the outside world, the damping layer itself strains to dissipate the energy generated by the vibration into thermal energy, thus achieving passive control [7]. This method is low-cost, stable, and effective for high-frequency vibration areas. However, it is not effective for low-frequency vibration areas and cannot be controlled in time when the external environment changes [8]. The advent of active control makes up for this deficiency by using intelligent materials such as piezoelectric ceramics as a piezoelectric layer to be pasted onto the surface, enabling the interconversion of electrical and kinetic energy through the piezoelectric effect, effectively enabling the control of low-frequency vibrations [9,10]. The disadvantages are the significant reduction in effectiveness when the controller fails, the poor suppression of high-frequency vibrations, and the high control costs [11,12]. Therefore, integrated active and passive control can combine the advantages of the first two control methods, and the disadvantages compensate for each other [13,14], as proposed by Baz [15] with the active constrained layer damping (ACLD) model. The difference from the PCLD structure is the replacement of the uppermost constraint layer with a piezoelectric layer [16,17]. Piezoelectric ceramics are usually used as the piezoelectric layer. Piezoelectric ceramics are suitable for use in structures such as plates, shells, and beams because of the small excitation required and the fast response time [18]. Figure 2 shows a typical ACLD structure.

The establishment of a mathematical model of the structure is a prerequisite for vibration control, and, currently, the standard methods are analytical, numerical, and experimental [19,20]. For a system with a relatively simple structure and relatively regular shape, the analytical method can be used. For complex structures, numerical methods should be used, in which the finite element method (FEM) is a common modeling method. The experimental method can be used to verify the accuracy of the models established by the analytical method and numerical method, and can also be used to describe the model characteristics of complex structures with the principle of system identification [21,22]. The model characteristics of the viscoelastic layer (VEM) should also be considered when modeling ACLD structures, where standard models include: complex constant shear modulus models, modal strain energy models, anelastic displacement field (ADF) models, and Golla–Hughes–McTavish (GHM) models. The complex constant shear modulus model is simple in structure, but it cannot reflect the phenomenon that the physical parameters of viscoelastic materials change with frequency. The modal strain energy model will produce large errors when the structure frequency is dense. Both the GHM model and ADF model describe the mechanical properties of viscoelastic materials in the frequency domain through a series of micro-oscillator terms, which can be effectively combined with FEM modeling methods to form a second-order differential equation [23,24]. However, the two models will introduce dissipative degrees of freedom in the use process, so that the dimension of degrees of freedom of the system is too large and must be used together with the order reduction method. Common model reduction methods include the GUYAN condensation method, the high-precision dynamic condensation method, the modal reduction method, the balanced reduction method, the robust reduction method, etc. The models can also be divided into physical order reduction and state-space order reduction. Physical order reduction can greatly reduce the degrees of freedom of the system, but it cannot guarantee the observability and controllability of the reduced order system; state-space order reduction can ensure the observability and controllability of the reduced system, but it is not suitable for systems with too high degrees of freedom [25,26]. Therefore, two order reduction methods are usually used together. Cao YQ [21] used the ADF model to characterize the relationship between the shear modulus of viscoelastic materials and temperature and used the finite element method to model ACLD beam and plate structures. Lu [23] introduced the GHM model to model the smart constrained layer damping (SCLD) thin plate structure and then obtained the decentralized subsystem control model through balanced order reduction and complex mode truncation. Li [27] established an experimental system for the ACLD beam structure and established the dynamic equation of the cantilever beam by using the finite element method, combining the piezoelectric equation, and introducing the complex constant modulus damping characteristics. Ray [28], based on the constitutive equations of piezoelectric materials and viscoelastic materials, deduced the dynamic equations of the partially covered ACLD cylindrical shell structure using the energy method. Huang and Mao [29] used the modal method and Hamilton principle to establish the dynamic equations of piezoelectric sandwich plates and solved them using the Rayleigh–Ritz method.

Common active control methods include proportional–integral differential (PID) control, linear quadratic optimal control (LQR), boundary control, robust control, etc. PID control is simple and easy to implement, but it is difficult to obtain the optimal gain. LQR control requires a higher accuracy for the model, all states can be fed back, resulting in a theoretically optimal feedback gain matrix, and robust control increases the stability of the system and resistance to disturbances. Li [30] proposed a hybrid PID–FXLMS algorithm combining the feedback FXLMS algorithm with a conventional PID controller and applied it to improve the efficiency of piezoelectric cantilever beam vibration control. Lu [31] designed a modal controller and modal state estimator in order to solve the full-state feedback problem, which made the system more suitable for real-time control. Zheng [32] designed a closed-loop control system with proportional (PD) feedback and derivative feedback based on sensor voltage and discussed the effect of control gain on the vibration suppression of ACLD cylindrical shell and plate structures. Zhang [33] designed a feedback-based LQG and feedforward FXLMS adaptive control composite controller to simulate and analyze the vibration problems of ACLD plate structures and compare the performance of the composite controller. Liu and Shi [34] designed a robust controller for active control of the first four orders of plate vibration using a robust control method. Liao [35] further applied the LQR control method to investigate the control force requirements for different configurations of ACLD structures and the response to the vibration effects. Lam [36] designed LQR controllers to optimize the controller parameters according to the characteristics of the actively constrained layer-damping structure. Baz [37] designed boundary controllers for ACLD beams, enabling the system to be kept globally stable in all vibration modes in boundary control.

Through the literature review, it is found that many scholars have conducted a lot of research in this field, but there are still some deficiencies, such as the ACLD sandwich board accurate modeling problem. For the active control of the ACLD structure, parameter selection in the LQR controller is always a hot issue, and the parameter optimization of the piezoelectric plate needs further study. Therefore, we conducted the following work, as described in this paper. In Section 2, modeling was performed for the ACLD sandwich plate based on the FEM method, and dynamic equations under different boundary conditions were obtained. In Section 3, the observability and controllability systems were obtained by the high-precision dynamic condensation and internal balance method. In Section 4, the accuracy of the models established by the method in this paper was verified, respectively, before and after order reduction. In Section 5, the control effect of the system under different boundary conditions and different incentives was explored through simulation analysis. The parameter optimization of the controller and the piezoelectric plate is discussed from the performance and cost points of view.

## 2. Finite Element Dynamic Model

**Figure 3:**
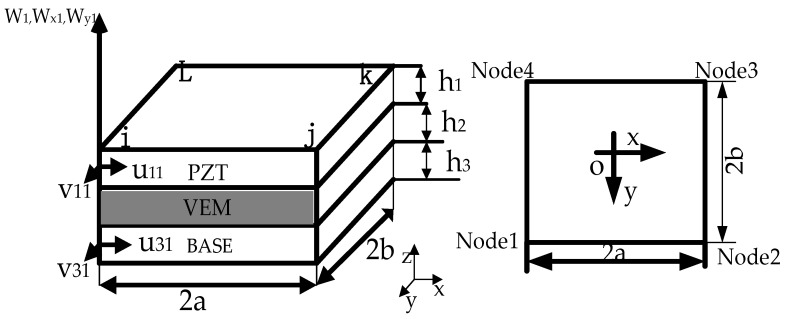
Schematic diagram of ACLD sandwich plates unit.

In this model, the object is first divided into several small units, as in Figure 3. Each unit has four nodes, and each node has seven degrees of freedom, respectively: x- and y-directional displacement within the piezoelectric plane, x- and y-directional displacement within the base plane, transverse displacement along the z-direction of the overall structure of the unit and the unit around the x- and *y*-axis direction of rotation, in turn, expressed as $v_{1}$,$u_{3}$,$v_{3}$,w,$w_{x}$,$w_{y}$, so a rectangular unit has 28 degrees of freedom. The modeling using the finite element method satisfies the seven assumptions mentioned in the literature [3].

### 2.1. Geometric Deformation Relationship between Layers of ACLD Structure

**Figure 4:**
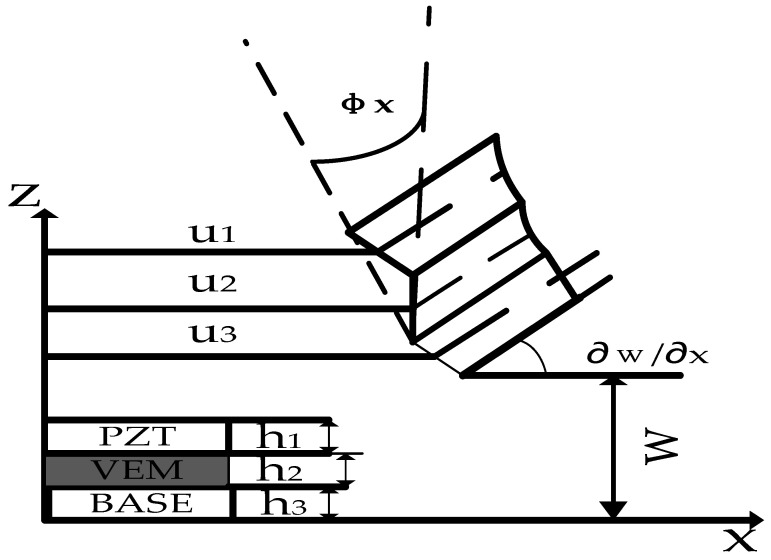
Plate deformation displacement diagram.

According to the assumption conditions and element freedom analysis, Figure 4 shows the coupled geometric deformation relationship of the ACLD plate element. The displacement of the viscoelastic layer in the *x*-axis direction and *y*-axis direction is as follows:

(1) $$u_{2}=\frac{1}{2}[(u_{1}+u_{3})+\frac{h_{1}-h_{3}}{2}\frac{\partial_{w}}{\partial_{x}}],v_{2}=\frac{1}{2}[(v_{1}+v_{3})+\frac{h_{1}-h_{3}}{2}\frac{\partial_{w}}{\partial_{x}}]$$

The shear strain generated by the viscoelastic layer rotating around the x- and y-axes is:

(2) $$\beta_{x}=\frac{u_{1}-u_{3}}{h_{2}}+\frac{d}{h_{2}}\frac{\partial_{w}}{\partial_{x}},\beta_{y}=\frac{v_{1}-v_{3}}{h_{2}}+\frac{d}{h_{2}}\frac{\partial_{w}}{\partial_{x}}$$

where $h_{1},\;h_{2},\;h_{3}$ represents the thickness of the piezoelectric layer, viscoelastic layer, and base layer, respectively, $d=\frac{h_{1}+h_{3}}{2}+h_{2}$.

### 2.2. Shape Function of ACLD Structure

As shown in Figure 3, the unit is rectangular in shape, and the size is 2a × 2b. Each element has four nodes (I, J, K, L), and each node has seven degrees of freedom, assuming that the displacement vector of node degrees of freedom is:

$$\{\Delta_{i}\}={\left[\begin{array}{ccccccc} u_{1i} & v_{1i} & u_{3i} & v_{3i} & w_{i} & w_{xi} & w_{yi} \end{array}\right]}^{T}$$

The displacement vector of the ACLD board element is:

$$\{U^{e}\}={\left\{\begin{array}{cccc} \Delta_{1} & \Delta_{2} & \Delta_{3} & \Delta_{4} \end{array}\right\}}^{T}$$

According to node displacement mode:

$$u_{1}=a_{1}+a_{2}x+a_{3}y+a_{4}xy,\;v_{1}=a_{5}+a_{6}x+a_{7}y+a_{8}xy$$

(3) $$u_{3}=a_{9}+a_{10}x+a_{11}y+a_{12}xy,v_{3}=a_{13}+a_{14}x+a_{15}y+a_{16}xy\;w=a_{17}+a_{18}x+a_{19}y+a_{20}x^{2}+a_{21}xy+a_{22}y^{2}+a_{23}x^{3}+a_{24}x^{2}y+a_{25}xy^{2}+a_{26}y^{3}+a_{27}x^{3}y+a_{28}xy^{3}\;w_{x}=\frac{\partial_{w}}{\partial_{x}};w_{y}=-\frac{\partial_{w}}{\partial_{y}}$$

where $a_{1},a_{2},\ldots .a_{28}$ are determined by the displacement vectors of 28 degrees of freedom of the four nodes of the element. Therefore, the displacement position of any point in the ACLD plate element can be obtained by interpolation of the element node displacement vector:

(4) $$\Delta ={\left[\begin{array}{ccccccc} u_{1} & v_{1} & u_{3} & v_{3} & w & w_{x} & w_{y} \end{array}\right]}^{T}=NU^{e}$$

where $N={\left[\begin{array}{ccccccc} N_{1} & N_{2} & N_{3} & N_{4} & N_{5} & N_{6} & N_{7} \end{array}\right]}^{T}$, respectively, corresponds to the spatial interpolation vector (shape function) of $\begin{array}{ccccccc} u_{1} & v_{1} & u_{3} & v_{3} & w & w_{x} & w_{y} \end{array}$. Substituting the shape function matrix obtained above into Equations (1) and (2), we can obtain the shape function matrix of the viscoelastic layer $u_{2}$,$v_{2}$ as follows:

(5) $$N_{8}=\frac{1}{2}[(N_{1}+N_{3})+\frac{h_{1}-h_{3}}{2}(-N_{7})],N_{9}=\frac{1}{2}[(N_{2}+N_{4})+\frac{h_{1}-h_{3}}{2}(N_{6})]$$

The shape function matrix of the shear strain of the viscoelastic layer is:

(6) $$N_{10}=\frac{1}{h_{2}}[(N_{1}-N_{3})-(\frac{h_{1}+h_{3}+2h_{2}}{2})(N_{7})],N_{11}=\frac{1}{h_{2}}[(N_{2}-N_{4})-(\frac{h_{1}+h_{3}+2h_{2}}{2})(N_{6})]$$

### 2.3. ACLD Structural Dynamics Equation

According to the classical plate theory, the virtual work performed by the kinetic energy, potential energy, and piezoelectric strain of each layer of the element thin plate can be derived by using the energy method. See Appendix A for details on obtaining the interlayer mass matrix, stiffness matrix, and piezoelectric matrix. Using Hamilton’s principle, the dynamic equation of the thin plate element is:

(7) $$M^{e}{\ddot{U}}^{e}+K^{e}U^{e}+GK_{\beta 2}^{e}U^{e}=F_{{}^{d}}^{e}+F_{c}^{e}$$

where $M^{e}=M_{1}^{e}+M_{2}^{e}+M_{3}^{e}$,$K^{e}=K_{1}^{e}+K_{2}^{e}+K_{3}^{e}$, *G* is the shear modulus of the viscoelastic layer material, $K_{\beta 2}^{e}$ is the shear stiffness matrix of the viscoelastic layer, $F_{{}^{d}}^{e}$ is the excitation force, and $F_{c}^{e}$ is the control force.

According to the finite element modeling method and considering its boundary constraints, all the element matrix groups were integrated into the total system matrix through the conventional set method, and the total dynamic equation of the system is derived as follows:

(8) $$M\ddot{X}+KX+GK_{\beta 2}X=F_{d}+F_{c}$$

where *M*, *K*, $K_{\beta 2}$, $F_{d}$, and $F_{c}$, respectively, represent the total mass matrix of the system, the total elastic stiffness matrix of the system, the shear stiffness matrix of the viscoelastic layer, the excitation force matrix, and the piezoelectric control force matrix.

Considering that the base layer has elastic damping, the proportional damping is expressed as:

(9) $$D=aM_{3}+bK_{3}$$

where $M_{3}$ and $K_{3}$ are the total mass matrix and total stiffness matrix of the base slab, and a and b are the mass and stiffness damping ratio coefficients.

*G* in Equation (8) is a function, not a constant term, and therefore a nonlinear equation. It is usually necessary to perform multiple iterative solutions. The model damping accuracy was also low, so the GHM damping model was introduced to characterize the mechanical properties of the viscoelastic layer. The GHM model uses the micro-vibrator term to characterize the viscoelastic material properties, and when the number of oscillators is N, Equation (8) after the Laplace transform is:

(10) $$\left(Ms^{2}+Ds+K+K_{\beta 2}G^{\infty }\left[1+\sum_{i=1}^{n}a_{i}\frac{s^{2}+{\hat{\xi }}_{i}{\hat{w}}_{i}s}{s^{2}+2{\hat{\xi }}_{i}{\hat{w}}_{i}s+{\hat{w}}_{i}{}^{2}}\right]\right)x(s)=F_{d}(s)+F_{c}(s)$$

where $G^{\infty }$ is the steady-state value of the relaxation function, and each oscillator term is a rational function containing three normal numbers ($a_{i}$,${\hat{\xi }}_{i}$,${\hat{w}}_{i}$). For the GHM model with N-order oscillator terms, the parameters to be determined are 3N + 1.

When dissipative degrees of freedom coordinates are introduced, we have:

(11) $$\hat{z}(s)=\frac{{\hat{w}}^{2}}{s^{2}+2\hat{\xi }\hat{w}s+{\hat{w}}^{2}}x(s)$$

The simultaneous Equations (8)–(11), an inverse Laplace transform, and boundary conditions were introduced to obtain the total dynamic equation of the ACLD structure as follows:

(12) $$\bar{M}\ddot{X}+\bar{D}\dot{X}+\bar{K}X={\bar{F}}_{d}+{\bar{F}}_{c}$$

where

$$\bar{M}=\left[\begin{array}{cccc} M & 0 & \cdots & 0\\ 0 & \frac{\alpha_{1}}{{\overset{⌢}{w}}_{1}{}^{2}}\Lambda & 0 & 0\\ \vdots & 0 & \ddots & \vdots \\ 0 & 0 & \cdots & \frac{\alpha_{N}}{{\overset{⌢}{w}}_{N}{}^{2}}\Lambda \end{array}\right],\bar{D}=\left[\begin{array}{cccc} D & 0 & \cdots & 0\\ 0 & \frac{2\alpha_{1}{\overset{⌢}{\xi }}_{1}}{{\overset{⌢}{w}}_{1}^{2}}\Lambda & 0 & 0\\ \vdots & 0 & \ddots & \vdots \\ 0 & 0 & \cdots & \frac{2\alpha_{N}{\overset{⌢}{\xi }}_{N}}{{\overset{⌢}{w}}_{N}^{2}}\Lambda \end{array}\right],{\bar{F}}_{d}=\left[\begin{array}{c} F_{d}\\ 0\\ \vdots \\ 0 \end{array}\right],{\bar{F}}_{c}=\left[\begin{array}{c} F_{c}\\ 0\\ \vdots \\ 0 \end{array}\right]$$

$$\bar{K}=\left[\begin{array}{cccc} K+G^{\infty }R_{v}\Lambda_{v}R_{v}{}^{T}\left(1+\sum_{i=1}^{n}a_{k}\right) & -a_{1}R_{v}G^{\infty }\Lambda_{v} & \cdots & -a_{N}R_{v}G^{\infty }\Lambda_{v}\\ -a_{1}{\left(R_{v}G^{\infty }\Lambda_{v}\right)}^{T} & a_{1}G^{\infty }\Lambda_{v} & 0 & \vdots \\ \vdots & 0 & \ddots & 0\\ -a_{N}{\left(R_{v}G^{\infty }\Lambda_{v}\right)}^{T} & & 0 & a_{N}G^{\infty }\Lambda_{v} \end{array}\right],X=\left[\begin{array}{c} X\\ Z_{1}\\ \vdots \\ Z_{N} \end{array}\right]$$

As the structure of the locally covered ACLD cell will be studied subsequently, it will result in the shear stiffness matrix $K_{\beta 2}$ containing stiffness information to avoid errors in the model, and an eigenvalue decomposition of $K_{\beta 2}$ is required to remove eigenvalues with zero and negative values.

(13) $$K_{\beta 2}=R_{v}\Lambda_{v}R_{v}^{T}$$

$R_{V}$ is the diagonal matrix formed by the positive eigenvalue of the viscoelastic shear stiffness matrix $K_{\beta 2}$ and $\Lambda_{v}$ represents the corresponding orthogonal eigenvector as the matrix of the column. After using the GHM model, the dynamic equations become ordinary second-order linear equations, which are convenient for solving the structural eigenvalue problem and establishing an effective model for the subsequent active control. Due to the introduction of dissipative degrees of freedom, the dimension of degrees of freedom of the system increases, the observability and controllability of the system cannot be guaranteed, the control cost increases, and the control stability decreases, so a model reduction was required.

## 3. Model Reduction

In order to make the model applicable to various controller designs, this paper adopted the joint order reduction method of dynamic condensation in physical space and state space internal balance. The degree of freedom of the system was significantly reduced and had good controllability and observability.

### 3.1. High Precision Dynamic Condensation Method

The following shows Equation (12) rewritten in chunked matrix form:

(14) $$\left[\begin{array}{cc} M_{mm} & M_{ms}\\ M_{sm} & M_{ss} \end{array}\right]\left[\begin{array}{c} {\ddot{X}}_{m}\\ {\ddot{X}}_{s} \end{array}\right]+\left[\begin{array}{cc} D_{mm} & D_{ms}\\ D_{sm} & D_{ss} \end{array}\right]\left[\begin{array}{c} {\dot{X}}_{m}\\ {\dot{X}}_{s} \end{array}\right]+\left[\begin{array}{cc} K_{mm} & K_{ms}\\ K_{sm} & K_{ss} \end{array}\right]\left[\begin{array}{c} X_{m}\\ X_{s} \end{array}\right]=\left[\begin{array}{c} F_{cm}\\ F_{cs} \end{array}\right]$$

where m and s represent the main degrees of freedom and the secondary degrees of freedom of the system, respectively.

The dynamic condensation matrix between the main and auxiliary degrees of freedom of the structure is: $R=K_{ss}^{-1}[(M_{sm}+M_{ss}R)M_{R}^{-1}K_{R}-K_{sm}]$. After (*i*) iterations, the dynamic condensation matrix is: $R^{i+1}=K_{ss}^{-1}[(M_{sm}+M_{ss}R^{i})(M_{R}^{i}{)}^{-1}K_{R}^{i}-K_{sm}]$, where the initial value of *R* is $R^{0}=K_{ss}^{-1}K_{sm}$. Similar to *R*, the final condensation matrices $M_{R},D_{R},K_{R}$ can also be obtained by iteration as follows:

(15) $$M_{R}^{i}=M_{mm}+{\left(R^{i}\right)}^{T}M_{sm}+{\left(R^{i}\right)}^{T}M_{ss}R^{i}+M_{ms}R^{i}$$

(16) $$D_{R}^{i}=D_{mm}+{\left(R^{i}\right)}^{T}D_{sm}+{\left(R^{i}\right)}^{T}D_{ss}R^{i}+D_{ms}R^{i}$$

(17) $$K_{R}^{i}=K_{mm}+{\left(R^{i}\right)}^{T}K_{sm}+{\left(R^{i}\right)}^{T}K_{ss}R^{i}+K_{ms}R^{i},F_{CR}^{i}=F_{cm}+{\left(R^{i}\right)}^{T}F_{cs}$$

In this paper, the *X* and *Y* displacements in the piezoelectric layer and base layers in the ACLD structure and the z displacement in the structure were selected as the principal degrees of freedom, and the dissipative degrees of freedom and the rest of the physical degrees of freedom were taken as the second degrees of freedom. The final kinetic equation after degradation is:

(18) $$M_{R}^{i}\overset{¨}{X_{m}}+D_{R}^{i}\dot{X_{m}}+K_{R}^{i}X_{m}=F_{CR}^{i}$$

### 3.2. Balanced Order Reduction Method

After dynamic condensation and order reduction, a model with a lower degree of freedom can be obtained, but the controllability and observability of the system model will be reduced. At the same time, after the system was converted to the state space equation, the dimension of the system was still significant, the control was unstable, and the control cost was too high. Therefore, it was necessary to perform order reduction in state space, remove some weakly observable and weakly controllable modes, and rewrite Equation (18) to the equation of state in state space as:

(19) $$\{\begin{array}{c} \overset{}{x=A\dot{x}+Bu}\\ y=Cx+Du \end{array}$$

where *A*, *B*, and *C* are, respectively, represented as the state matrix, input matrix, and input matrix of the system. The controllability GRAMMIANS matrix of the system is: $W_{c}=\int_{0}^{\infty }e_{A\tau }{}^{T}B^{T}Be_{A\tau },$ and the observability GRAMMIANS matrix of the system is: $W_{o}=\int_{0}^{\infty }e_{A\tau }{}^{T}C^{T}Ce_{A\tau }d\tau .$ Then, defining a non-singular transformation matrix *R* and performing a balanced transformation gives $\bar{x}$ = *Rx*. Substituting this transformation into Equation (19) to obtain a new controllability and observability matrix produces:

(20) $$\bar{W_{c}}=RW_{c}R^{-1},\bar{W_{o}}=RW_{o}R^{-1}$$

A suitable transformation matrix *R* can cause $\bar{W_{c}}=\bar{W_{o}}=diag\left(g\right)$, at which point the system is in equilibrium. Here, diag (g) is the singular value diagonal matrix of the matrix, reflecting the system’s modal observability and controllability index. It was then required to sort the elements in g and delete the smaller values. These smaller values have little impact on the system and are not observable or controllable.

We then rewrote the state variable as: $\bar{x}={\left[{\bar{x}}_{r}\;{\bar{x}}_{d}\right]}^{T}$, with ${\bar{x}}_{r}$ as the amount of retention and ${\bar{x}}_{d}$ as the amount of deletion, and the equation of the state of the system is:

(21) $$\left[\begin{array}{c} \dot{{\bar{x}}_{r}}\\ \dot{{\bar{x}}_{d}} \end{array}\right]=\left[\begin{array}{cc} \bar{A_{rr}} & \bar{A_{dr}}\\ \bar{A_{rd}} & \bar{A_{dr}} \end{array}\right]\left[\begin{array}{c} {\bar{x}}_{r}\\ {\bar{x}}_{d} \end{array}\right]+\left[\begin{array}{c} {\bar{B}}_{r}\\ {\bar{B}}_{d} \end{array}\right]u,y=[{\bar{C}}_{r}{\bar{C}}_{d}]\left[\begin{array}{c} {\bar{x}}_{r}\\ {\bar{x}}_{d} \end{array}\right]+\;\bar{D}u.$$

Let ${\bar{x}}_{d}=0$, that is, delete the state variable, and the new state equation after balance reduction can be obtained as follows:

(22) $$\{\begin{array}{c} \dot{{\bar{x}}_{r}}=\bar{A_{rr}}{\bar{x}}_{r}+{\bar{B}}_{r}u\\ y={\bar{C}}_{r}{\bar{x}}_{r}+\bar{D}u \end{array}$$

Compared with Equation (19), Equation (22) further reduces the dimension of the model’s degree of freedom and ensures the observability and controllability of the model system.

## 4. Finite Element Model Verification

In this section, the accuracy of the original model and the accuracy of the model after joint reduction were verified through the analysis of numerical examples. The optimal number of discrete elements of the finite element model was obtained through the convergence test. The geometric and material parameters of each layer are as follows.

Piezoelectric layer: $p_{1}=7450kg/m^{3},E_{1}=74.5GPa,\mu_{1}=0.32,h_{1}=0.5mm$.

Damping layer: $p_{2}=789kg/m^{3},\mu_{2}=0.3,h_{2}=1mm$,

Base layer: $p_{3}=2800kg/m^{3},E_{3}=70GPa,\mu_{3}=0.3,h_{3}=0.8mm$;

Base size: The total length is 0.2 m and the width is 0.1 m. Piezoelectric constant: $d_{11}=d_{22}=2.8\times 10^{-9}$

GHM model parameters were taken according to the literature [20]: $G^{\infty }$ = 5.542 × 10^5^,

$a_{i}$ = [3.96, 65.69, 1.447], ${\overset{⌢}{w}}_{i}$ = [8.962 × 10^5^, 9.278 × 10^5^, 7.613 × 10^5^], ${\overset{⌢}{\xi }}_{i}$ = [148, 12.16, 810.4], where N = 3, there are 3N + 1 = 10 parameters.

### 4.1. Finite Element Model Element Convergence Test

The convergence of the elements is an important property of the finite element method, which is directly related to whether the convergence solution can be obtained and whether the appropriate number of elements can be used to obtain a satisfactory solution. The purpose of the convergence test is to test the influence of the number of finite element elements on the calculation results. When the number of elements increases and the result does not change significantly (i.e., tends toward a stable value), the calculation result is considered to be convergent. If the number of finite element elements increases, the calculation results always fluctuate greatly, which means that the element does not converge and a reasonable finite element solution cannot be obtained.

**Figure 5:**
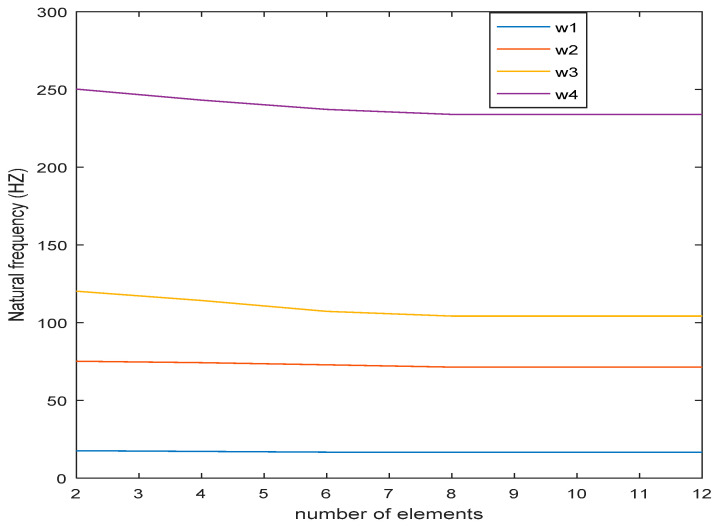
Chart of influence of element number on natural frequency of ACLD thin plate.

To investigate the convergence of the finite element model in this paper, that is, to discuss the discrete effect of the number of units on the result is to discuss how much is needed, it was appropriate to discretize the unit. Because too few units may lead to non-convergence in results, too many elements will lead to an increase in the workload of element assembly and equation solving. Therefore, a simple numerical example was designed to study this problem. The ACLD plate structure was discretized by the four-node, 28-degree-of-freedom plate element derived in this paper, and the number of elements was two, four, six, eight, and ten. The calculated natural frequency changes with the number of elements are shown in Figure 5.

It can be seen from Figure 5 that this element has good convergence. When the number of elements is six, the calculation results of the natural frequency of the system begin to have an obvious convergence trend. When the number of elements is eight, the curve is basically unchanged and the convergence requirements can be considered.

### 4.2. Model Validation before Order Reduction

**Figure 6:**
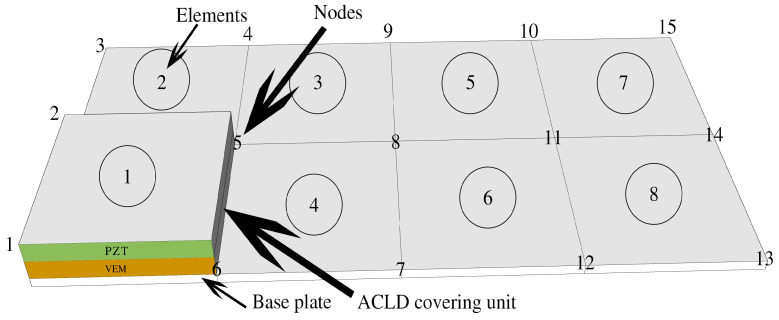
Finite element model of ALCD thin plate structure.

Under different boundary conditions, the modal parameters of the base plate and ACLD sandwich plate were calculated by using Ansys2021 finite element analysis software and Matlab2016 finite element program, respectively, and the correctness of the model was further verified by comparing with the modal experiments results from the literature [23,31,33]. The boundary conditions of the four sides of the plate are represented by letters, and their meanings are: C—fixed support and F—free. CFFF means that one side is fixed and the other three sides are free; CFCF means that two opposite edges are fixed and the other two edges are free; CCCC stands for four-sided fixed support. Figure 6 shows the finite element model of the ALCD thin plate structure. According to the convergence analysis results, the bottom plate was divided into eight elements and 15 nodes. The element type in the finite element model is SHELL63, an elastic plate and shell element, and a mesh contact was used between the plate and shell elements. The linear contact mode was adopted between the layers, and the materials of each layer were firmly pasted without relative sliding between layers. The ACLD unit was partially covered. The coverage position is represented by element 1 in the figure.

**Figure 7:**
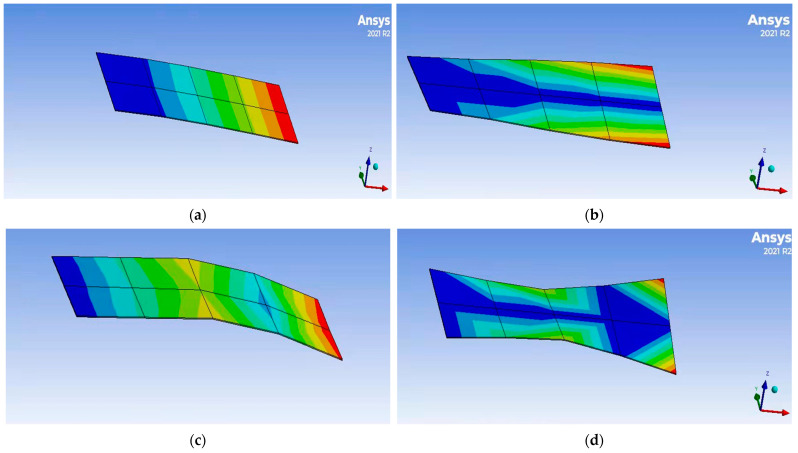
The first four modal shapes of cantilever plate (Ansys solution)**.** (**a**) First-order bending mode; (**b**) second-order torsional mode; (**c**) third-order bending mode; (**d**) fourth-order torsional mode.

**Figure 8:**
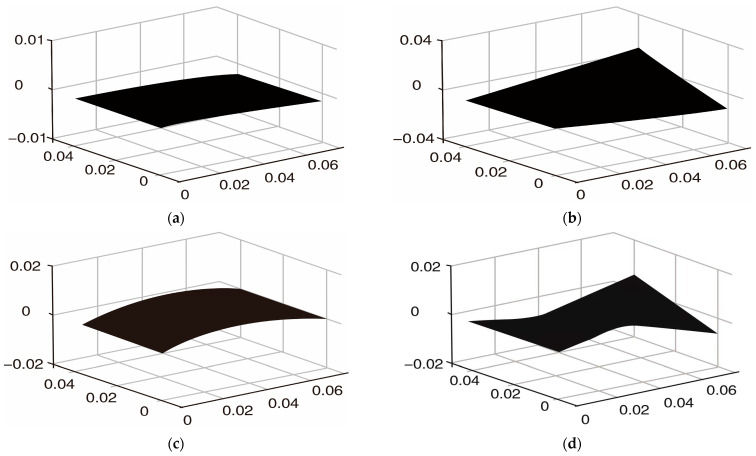
The first four modal shapes of cantilever plate (Matlab numerical solution). (**a**) First-order bending mode; (**b**) second-order torsional mode; (**c**) third-order bending mode; (**d**) fourth-order torsional mode.

It can be seen from Table 1 that the difference between the numerical solution obtained based on Matlab programming and the results of Ansys2021 software, references, and modal experiments is very small. It can be seen from Table 2 that the difference between the numerical solution of Matlab and the results of Ansys and references is also very small. Compared with the results in Table 1 and Table 2, the coverage of the piezoelectric layer and viscoelastic layer leads to a reduction in natural frequency, which is consistent with the actual results. The maximum error of the first four natural frequencies of the system is not more than 5%, and the unit of natural frequencies is Hz. Figure 7 shows the first four modal shapes of the cantilever plate obtained via Ansys2021 software. Figure 8 shows the first four modal shapes of the cantilever plate obtained with a Matlab numerical solution. Figure 7 and Figure 8 show that the mode of shape deformation of the cantilever plate is similar. Therefore, the above results show that the finite element modeling method in this paper is accurate.

### 4.3. Model Verification after Order Reduction

The full coverage ACLD structure model under one side fixed support was selected as the research object, and the natural frequencies before and after the model reduction were calculated through Matlab, the Bode diagram in the frequency domain and the pulse response diagram in the time domain were drawn, and the controllability and observability of the system were judged.

As shown in Table 3, the error of the first- to fourth-order natural frequencies of the model is less than 3% after two-order reduction methods are adopted, which has little impact on the natural characteristics of the system. As shown in Table 4, before the reduction, the system dimension was 210, the WC and WO matrices were not full of rank, and the system was uncontrollable and unobservable. After dynamic condensation, the system dimension was greatly reduced, but the observability matrix and controllability matrix were not full of rank. Next, we balanced the reduced order model, arranged the state variable diag (g) from small to large, and retained a value greater than 0.002. Finally, the system model only had six degrees of freedom, and the observability and controllability matrices had full rank. The vibration characteristics of the system were mainly determined by the first- to fourth-order modal characteristics, and the subsequent active control was mainly carried out around the low-frequency modes.

**Figure 9:**
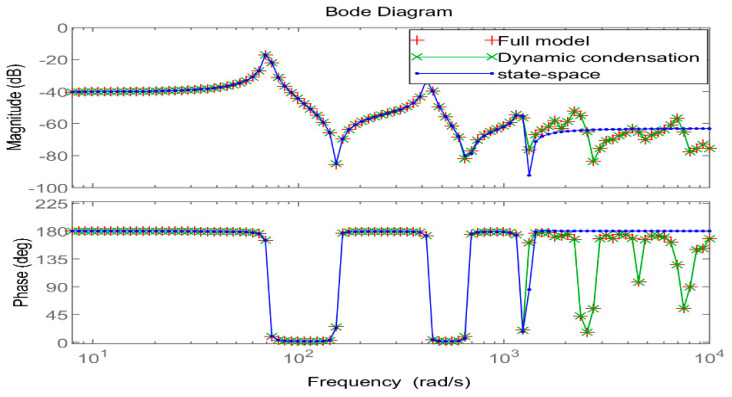
Frequency domain Bode diagram comparison before and after order reduction.

**Figure 10:**
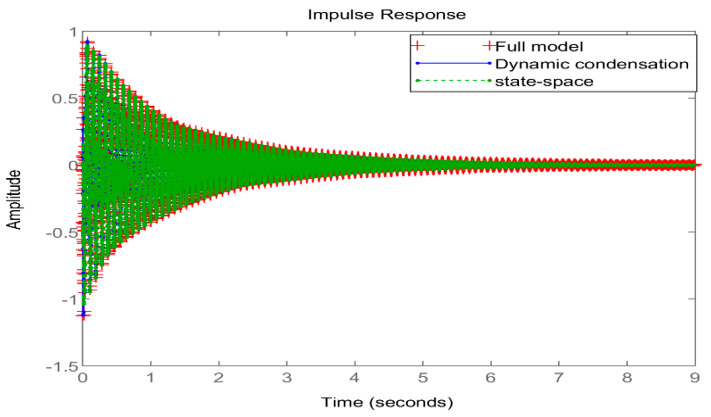
Comparison of time–domain impulse response diagrams before and after order reduction.

Figure 9 shows the Bode diagram of the system in the frequency domain. After the model was reduced twice, it could still accurately and reliably describe the dynamic characteristics of the original system in the low-frequency region. Figure 10 shows the time–domain response diagram of the original system under impulse excitation after two order reductions. From the degree of fitting of the time–domain response curve, the time–domain effect of the two order reductions on the system is small. To sum up, the order reduction method proposed in this paper is effective, which not only greatly reduces the degree of freedom of the system but also, ultimately, makes the system observable and controllable, providing favorable help for the design of an active controller.

## 5. Vibration Control and Parameter Optimization of ACLD Thin Plates

This chapter details the conduction of vibration control and parameter optimization for the reduced system model. The first section outlines the design of the LQR controller, the second section examines the control effect of the LQR controller on the system in different scenarios, the optimization of the parameters of the LQR controller is discussed in the third section, and the optimization of the parameters of the piezoelectric chip is discussed in the fourth section.

### 5.1. LQR Control

LQR control is the optimal control based on the state space expression, and the state space equation of the system was obtained, as shown in Equation (22):

(23) $$\{\begin{array}{c} \dot{{\bar{x}}_{r}}=\bar{A_{rr}}{\bar{x}}_{r}+{\bar{B}}_{r}u\\ y={\bar{C}}_{r}{\bar{x}}_{r}+\bar{D}u \end{array}$$

By designing the optimal feedback controller, the objective function *J* is minimized to:

(24) $${J=\int }_{0}^{\infty }\left(x_{r}^{T}Qx_{r}+u^{T}Ru\right)dt$$

where *Q* and *R* are, respectively, the output vector weighting matrix and the control vector weighting matrix, and u is the control voltage of the system [38,39].$u=-kx_{r},k=R^{-1}{\bar{B}}_{r}{}^{T}P.$

**Figure 11:**
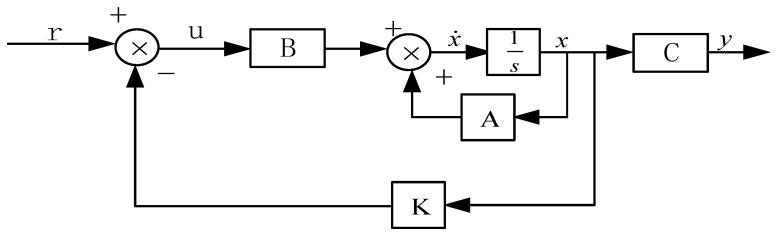
LQR controller block diagram.

*P* satisfies the Riccati equation: $P\bar{A_{rr}}+{\bar{A_{rr}}}^{T}p-p{\bar{B}}_{r}R^{-1}{\bar{B}}_{r}{}^{T}p+{\bar{C}}_{r}{}^{T}Q{\bar{C}}_{r}=0$. The state equation of the closed-loop system is: $\dot{x}=(\bar{A_{rr}}-{\bar{B}}_{r}K){\bar{x}}_{r}+{\bar{B}}_{r}u$. The purpose of the LQR controller design was to solve the Riccati equation. The essence of obtaining the control gain matrix *k* was to minimize the objective function *J*. The *Q* and *R* weighting matrices in the equation represent performance and cost, respectively. Different *Q* and *R* values will make the optimal control scheme of the system different. Therefore, it is necessary to select weighting coefficients reasonably according to their own needs [40,41]. As shown in Figure 11, the LQR controller block diagram further describes the control principle.

### 5.2. Vibration Control of ACLD Thin Plates

**Figure 12:**
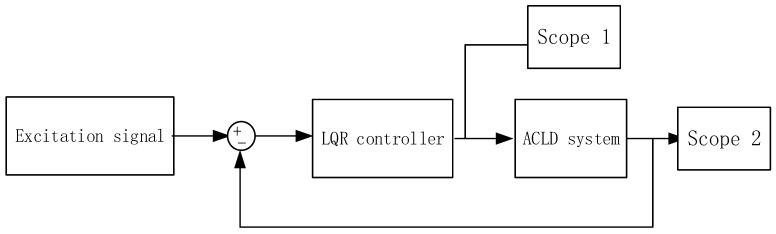
ACLD system control block diagram.

Two boundary conditions, CFFF and CFCF, were selected, and the coverage mode was to cover the piezoelectric patch locally. The effect of the system under LQR control was observed under pulse excitation and a limited-bandwidth white noise of 20–100 Hz. Where the impulse excitation represents the unit shock acting on the system at the initial time, Figure 12 shows the control block diagram of the ACLD system.

**Figure 13:**
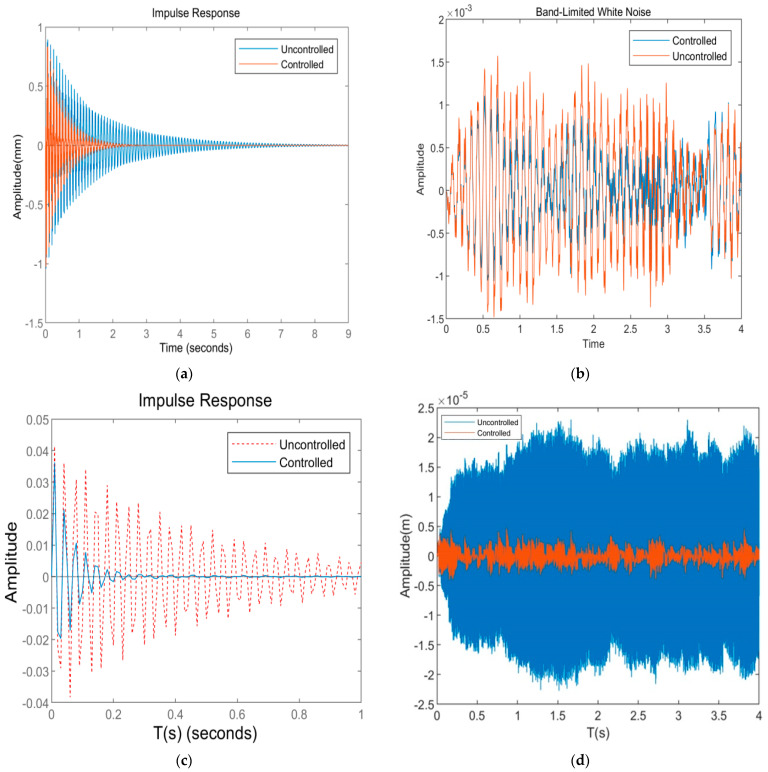
Response diagram before and after system control. (**a**) Response of CFFF plate under pulse excitation; (**b**) CFFF plate response under white noise; (**c**) response of CFCF plate under pulse excitation; (**d**) CFCF plate response under white noise.

Figure 13a shows that the maximum displacement of the CFFF plate without voltage is 0.871 mm, the convergence time is 5.51 s, the maximum displacement under the control of LQR is 0.751 mm, and the convergence time is 2.5 s. The maximum displacement reduction is 6.17%. Figure 13b shows that the root mean square value of the vibration response displacement of the CFFF plate is 0.876 × 10^−5^ m when no voltage is applied. Under the control of LQR, the root mean square value of the vibration response decreases to 0.574 × 10^−5^ m, with a decrease of 34.4%. Figure 13c shows that the maximum displacement of the CFCF plate is 0.042 mm without a voltage applied, and the convergence time is about 4.56 s. Under the control of LQR, the maximum displacement is 0.036 mm, the convergence time is 0.97 s, and the maximum displacement reduction is 16%. Figure 13d shows that the root mean square value of the vibration response displacement of the CFCF plate is 1.78 × 10^−5^ m when no voltage is applied. Under the control of LQR, the root mean square value of the vibration response decreases to 0.23 × 10^−5^ m, with a decrease of about 87%. The simulation shows that under different boundary conditions and excitation, the amplitude decreases greatly, the vibration converges rapidly, and the ACLD sandwich plate can achieve a high level of active control.

### 5.3. Parameter Optimization of LQR Controller

**Figure 14:**
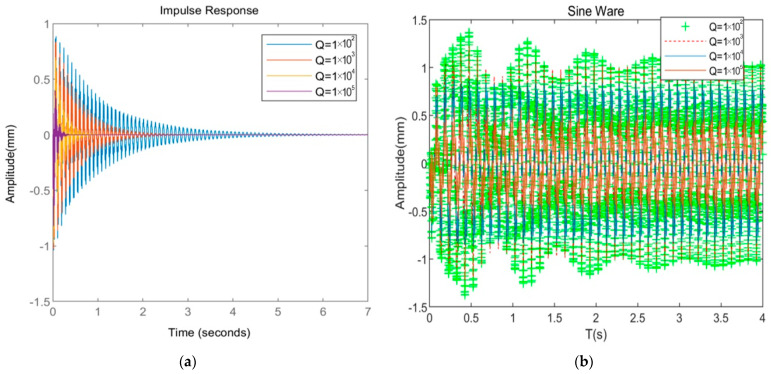
Comparison of system response with different Q-matrix coefficients. (**a**) System response under impulse excitation; (**b**) system response under sine wave.

**Figure 15:**
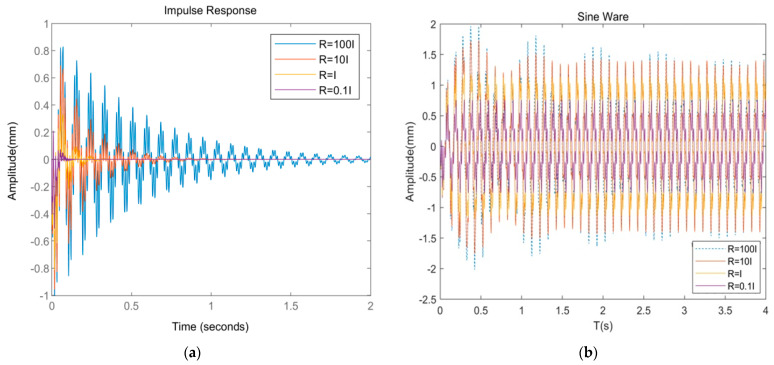
Comparison of system response with different R-matrix coefficients. (**a**) System response under impulse excitation; (**b**) system response under sine wave.

**Figure 16:**
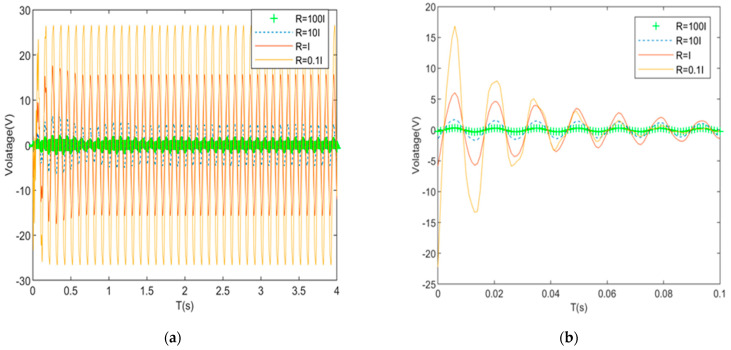
Voltage contrast required for control when R-matrix coefficient is different. (**a**) Required voltage under pulse excitation; (**b**) voltage required under sine wave.

The previous section verified that the LQR controller can achieve active control over the structure. This section explored the selection of *Q* and *R* weighting matrices in the controller. The *Q* and *R* matrices are set in the form of $Q=\alpha {\bar{C}}_{r}{}^{T}{\bar{C}}_{r},R=\beta I$, where α and β are the coefficients of the matrix and I is the unit matrix. The optimal scheme of *Q* and *R* matrices needs constant trial and simulation. Take the cantilever plate as an example, the simulation analysis was carried out by changing the coefficient of the weighting matrix under two different excitations. When the coefficient of the *Q* matrix changed, *R* = 100*I*. When the coefficient of the *R* matrix changed, the *Q* coefficient was 1 × 10^2^. The simulation results are shown in Figure 14, Figure 15 and Figure 16.

Figure 14a,b show the system response curve when the *Q*-matrix coefficient of the system changes under pulse excitation and sine wave, respectively. The specific values are shown in Table 5 and Table 6. In summary, when the *Q*-matrix coefficients take different values, the following conclusions exist under two different excitations: (1) As the *Q*-matrix coefficient increases, the amplitude of CFFF decreases, the convergence time decreases, and the control effect increases. (2) When the *Q*-matrix coefficient is from 1 × 10^2^ to 1 × 10^4^, the maximum amplitude ranges from 0.855 mm to 0.812 mm to 0.721 mm, and the root mean square value ranges from 0.842 mm to 0.701 mm. The decrease is not apparent, indicating that the *Q*-matrix coefficient is not the best at this time. When the *Q* coefficient is from 1 × 10^4^ to 1 × 10^5^, the maximum amplitude ranges from 0.721 mm to 0.391 mm, the root mean square value of the amplitude ranges from 0.701 mm to 0.328 mm, and the convergence time ranges from 2.51 s to 0.31 s. The average decline of the three indicators reached more than 75%. From the perspective of cost control and performance balance, the optimal *Q*-matrix coefficient suitable for this system is between 1 × 10^4^ and 1 × 10^5^.

Figure 15a and Figure 16a show that under pulse excitation, when the R-matrix coefficient is 100, the maximum amplitude is 0.812 mm, the convergence time is 2.02 s, and the maximum drive voltage is 0.81 V. When the R-matrix coefficient is 10, the maximum amplitude is 0.695 mm, the convergence time is 1.21 s, and the maximum driving voltage is 1.81 V. When the R-matrix coefficient is 1, the maximum amplitude is 0.351 mm, the convergence time is 0.41 s, and the maximum driving voltage is 5.85 V. When the R-matrix coefficient is 0.1, the maximum amplitude is 0.211 mm, the convergence time is 0.21 s, and the maximum driving voltage is 16.8 V. Figure 15b and Figure 16b show that under the sine wave, when the R-matrix coefficient is 100, the root of the square value of the amplitude is 1.61 mm, and the maximum driving voltage is 2.21 V. When the R-matrix coefficient is 10, the root mean square value of the steady-state response curve decreases to 1.53 mm, with a reduction of 4.9%, and the maximum driving voltage is 6.78 V. When the R-matrix coefficient is 1, the root mean square value of the steady-state response curve decreases to 0.89 mm, with a reduction of 44.7%, and the maximum driving voltage is 15.2 V. When the R-matrix coefficient is 0.1, the root mean square value of the steady-state response curve decreases to 0.58 mm, with a reduction of 63.1%, and the maximum driving voltage is 27.8 V.

To sum up, when the value of the R-matrix coefficient is different under two different excitations, the following rules exist: (1) As the R-matrix coefficient decreases, the amplitude of the cantilever plate decreases, the convergence time decreases, the control effect increases, and the required driving voltage increases. (2) When the coefficient of the R matrix decreases from 100 to 10 for the first time, the amplitude of the system does not decrease significantly, and the control effect is poor. The optimal coefficient of the R matrix is not in this range. When the coefficient decreases from 10 to 1 for the second time, the system amplitude decreases significantly. Considering the control cost and performance balance, the R-matrix coefficient applicable to this paper is between 10 and 1. If you want to obtain more accurate solutions, you can shorten the scope for further simulation.

The LQR controller parameter optimization problem should be discussed under the precise model and according to the designer’s needs. The above simulation content is a design scheme considering the balance of control cost and performance. When the designer is more concerned about the control performance, they can focus on the weight of the Q-matrix coefficient in the objective function J, followed by the R-matrix coefficient. If the control cost of a system is high, the designer can focus on the weight of the R-matrix coefficient in the objective function J to obtain a satisfactory scheme.

### 5.4. Piezoelectric Plate Parameter Optimization

**Figure 17:**
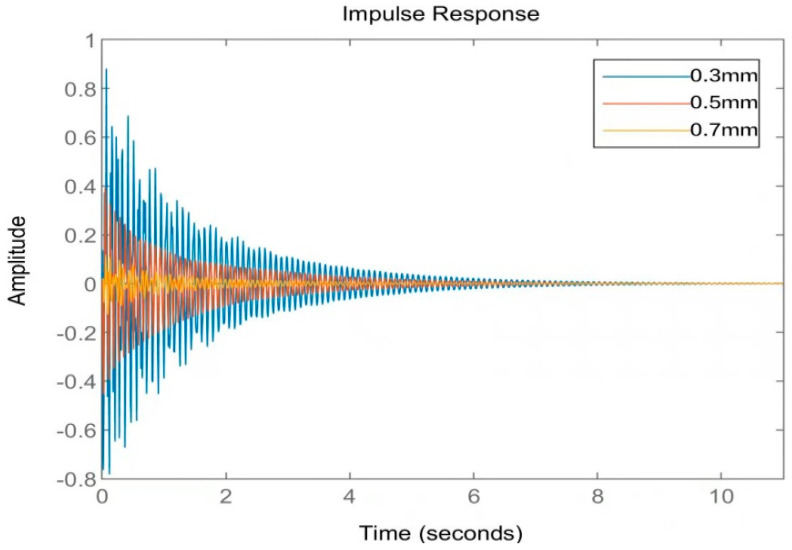
System response (no voltage applied) when piezoelectric sheets are of different thicknesses.

**Figure 18:**
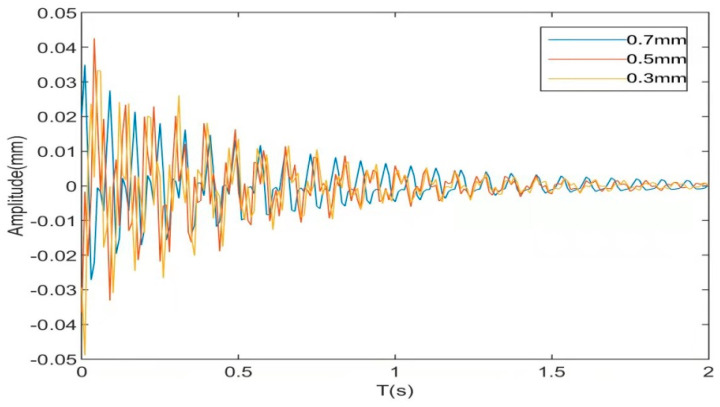
System response at different thicknesses of piezoelectric sheets (driving voltage applied).

**Figure 19:**
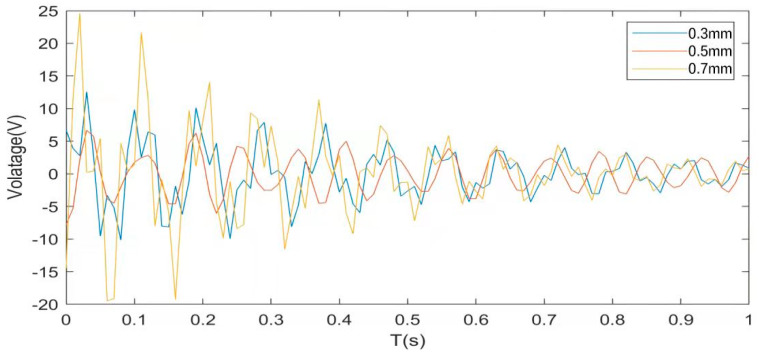
Control the required voltage when the piezoelectric sheet is of different thicknesses.

The structure relies on the piezoelectric effect of the piezoelectric chip to achieve active control. The main factor that affects the control effect is the various parameters of the piezoelectric chip. The thickness of the piezoelectric plate has a great influence on the vibration characteristics of the CFFF plate under pulse excitation [42,43]. The controller parameters do not change, and the simulation results are shown in Figure 17, Figure 18 and Figure 19.

Figure 17 shows that the maximum amplitude of the cantilever plate is 0.834 mm and the convergence time is 7.03 s when the thickness of the piezoelectric plate is 0.3 mm without applying the driving voltage. When the thickness of the piezoelectric sheet is 0.5 mm, the maximum amplitude is 0.395 mm, and the convergence time is 5.91 s. When the thickness of the piezoelectric sheet is 0.7 mm, the maximum amplitude is 0.176 mm, and the convergence time is 4.41 s due to the large material density of the piezoelectric layer. When the thickness of the piezoelectric sheet increases from 0.3 mm to 0.7 mm, the mass matrix of the system increases significantly, reducing the natural frequency of the system and also effectively suppressing the structural vibration. Passive control by only changing the thickness of the piezoelectric sheet has the following advantages and disadvantages. The advantages are that the maximum amplitude is obviously reduced, the system has a good suppression effect in the high-frequency vibration area, and the control cost is low. The disadvantages are as follows: (1) The convergence time decreases slowly, and the control effect is not good in the low-frequency vibration area of the system; (2) The control effect is limited, and blindly increasing the thickness will lead to excessive quality in the system.

Figure 18 and Figure 19 show that, under the pulse excitation and after the drive voltage is applied to the system, the maximum amplitude of the CFFF plate is 0.048 mm, the convergence time is 3.1 s, and the maximum drive voltage is 11.8 V when the piezoelectric thickness is 0.3 mm. When the thickness of the piezoelectric sheet is 0.5 mm, the maximum amplitude is −0.039 mm, the convergence time is 2.7 s, and the maximum driving voltage is −8.02 V. When the thickness of the piezoelectric sheet is 0.7 mm, the maximum amplitude is 0.034, the convergence time is 2.2 s, and the maximum driving piezoelectric is 24.8 V. All of the above simulations were performed without changing the LQR controller parameters, from which the following conclusions can be drawn: (1) With the increase in the thickness of the piezoelectric sheet, the active control effect of the sandwich plate is continuously enhanced, but the control effect is not apparent from the perspective of the maximum amplitude and convergence time, and the required voltage cost increases significantly. (2) The system performs best when the thickness of the piezoelectric sheet is 0.5 mm. Compared with a thickness of 0.3 mm, the active control effect is slightly better, and the cost is slightly lower. Compared with a thickness of 0.7 mm, the active control effect is slightly worse, and the cost is far lower than 24.8 V. To sum up, designers should consider their own system, application scenarios, performance, and cost comprehensively when choosing the thickness of piezoelectric sheets. In pursuit of thinness, some control performance must be discarded. If you want to pursue the ultimate control effect, you must increase the control cost and increase the overall quality of the system.

## 6. Conclusions

The dynamic model of active constrained layer damping thin plate structure under three different boundary conditions was established through the finite element method. A GHM damping model was used to characterize the dynamic characteristics of the viscoelastic layer, and the elastic damping of the base layer was expressed by proportional damping. The suitable model for LQR control was obtained through the joint order reduction method of dynamic condensation in physical space and equilibrium in state space. The parameters of the LQR controller and piezoelectric chip were optimized through simulation analysis. The research shows that:

(1) The finite element model established in this paper is accurate and feasible under different boundary conditions. The introduction of the GHM damping model and base proportional damping further improves the accuracy of the structural dynamic model.
(2) The GHM model introduces a large number of dissipative degrees of freedom, and the dimension of the finite element model is relatively large. The mathematical model obtained through the joint reduction method has a low degree of freedom and good controllability and observability. Whether in the time domain or frequency domain, the dynamic characteristics of the reduced model are unchanged. It can be directly used in system controller design.
(3) According to the simulation analysis, the reduced model can effectively suppress the vibration by using LQR control under different boundary conditions and different excitations. The Q and R weighting matrices will affect the control effect of the system. From the performance and cost balance, the optimal Q-matrix coefficient is found to be 1 × 10^4^ to 1 × 10^5^, and the R-matrix coefficient is between 10 and 1.
(4) When the voltage is not applied, the structure is controlled passively. Increasing the thickness of the piezoelectric sheet can effectively suppress the vibration in the high-frequency region, while the vibration in the low-frequency region is not significantly suppressed. When the driving voltage is applied and the thickness of the piezoelectric chip increases, the control effect of the system can be improved, but it is not obvious. On the contrary, the voltage cost increases significantly. Therefore, considering the control effect and cost balance, the most suitable thickness of the piezoelectric sheet for this system is 0.5 mm.

Therefore, the method provided in this paper is feasible and has a guiding role in solving the vibration problems caused by thin plates in engineering applications. This method can also be used to study the vibration of other sandwich structures in the future. As for the optimization of controller parameters, the algorithm can be introduced in follow-up research to optimize and obtain a more enhanced scheme.

## Figures and Tables

**Table 1:** Comparison of natural frequencies of substrates under different boundary conditions.

CFFF				CFCF			CCCC				
Mode	Matlab	Ansys	[33]	Test	Matlab	Ansys	Test	Matlab	Ansys	Test	Error
1	16.581	16.5635	16.446	16.312	107.49	107.4372	106.571	478.21	476.41	474.02	0.5%
2	71.386	71.1448	70.778	70.753	174.3	173.4320	171.242	618.27	616.26	613.61	1.2%
3	104.26	103.620	102.54	101.52	302.8	298.7831	298.721	872.39	868.53	863.46	2.0%
4	233.96	231.926	230.41	228.25	405.97	392.9264	398.414	1243.1	1234.6	1222.7	2.8%

**Table 2:** Comparison of natural frequencies of ACLD sandwich plates under different boundary conditions.

CFFF					CFCF		
Mode	Matlab	Ansys	[33]	Error	Matlab	Ansys	Error
1	16.121	16.021	16.754	0.67%	96.7274	96.3502	0.39%
2	69.212	68.232	70.729	1.46%	151.384	149.251	1.31%
3	100.11	97.411	98.940	2.41%	270.813	263.691	2.63%
4	221.25	213.35	230.41	3.58%	350.51	336.14	4.11%

**Table 3:** Comparison of natural frequencies before and after model order reduction.

Mode Order	Full Model	Dynamic Condensation	State-Space	Maximum Error
1	11.3050	11.2687	11.2591	0.1%
2	47.5475	47.4804	46.8541	1.3%
3	68.3629	68.4804	67.1951	1.6%
4	151.0806	150.0142	148.1251	1.9%

**Table 4:** Judgment indexes of controllability and observability before and after model reduction.

	Size (WC)	Rank (WC)	Size (WO)	Rank (WO)
Full model	210	66	210	71
Dynamic condensation	120	58	120	63
State-space	6	6	6	6

Note: Size () represents the matrix dimension, Rank () represents the rank of the matrix, WC represents the controllability matrix, and WO represents the observability matrix.

**Table 5:** System response parameters under impulse excitation.

*Q*-matrix coefficient	1 × 10^2^	1 × 10^3^	1 × 10^4^	1 × 10^5^
Maximum amplitude	0.855 mm	0.812 mm	0.721 mm	0.391 mm
Convergence time	5.34 s	4.42 s	2.51 s	0.31 s

**Table 6:** System response parameters under sine wave.

*Q*-matrix coefficient	1 × 10^2^	1 × 10^3^	1 × 10^4^	1 × 10^5^
Amplitude RMS value	0.842 mm	0.809 mm	0.701 mm	0.328 mm

## Data Availability

Data sharing not applicable.

## Appendix A

The kinetic energy of each layer of the thin plate and the mass matrix of each layer are as follows:

(A1) $$T_{i}{}^{\left(e\right)}=\frac{1}{2}m_{i}V_{i}^{2}=\frac{p_{i}h_{i}}{2}\int_{0}^{2a}\int_{0}^{2b}{\left(\frac{\partial w}{\partial t}\right)}^{2}+{\left(\frac{\partial u_{i}}{\partial t}\right)}^{2}+{\left(\frac{\partial v_{i}}{\partial t}\right)}^{2}dxdy=\frac{1}{2}{\left\{{\dot{X}}^{\left(e\right)}\right\}}^{T}\left[M_{i}{}^{\left(e\right)}\right]\left\{{\dot{X}}^{\left(e\right)}\right\}$$

(A2) $$M_{1}^{\left(e\right)}=p_{1}h_{1}\int_{0}^{2a}\int_{0}^{2b}\left(N_{1}^{T}N_{1}+N_{2}^{T}N_{2}+N_{5}^{T}N_{5}\right)dxdy$$

(A3) $$M_{2}^{\left(e\right)}=p_{2}h_{2}\int_{0}^{2a}\int_{0}^{2b}\left(N_{8}^{T}N_{8}+N_{9}^{T}N_{9}+N_{5}^{T}N_{5}\right)dxdy$$

(A4) $$M_{3}^{\left(e\right)}=p_{3}h_{3}\int_{0}^{2a}\int_{0}^{2b}\left(N_{3}^{T}N_{3}+N_{4}^{T}N_{4}+N_{5}^{T}N_{5}\right)dxdy$$

The dynamic potential energy of each layer of the thin plate and the stiffness matrix of each layer are as follows:

(A5) $$E_{i}^{\left(e\right)}=\int_{0}^{2a}\int_{0}^{2b}\int_{\frac{-h_{i}}{2}}^{\frac{h_{i}}{2}}\left[\sigma_{ix}\varepsilon_{ix}+\sigma_{iy}\varepsilon_{iy}+\tau_{ixy}\gamma_{ixy}\right]dxdydz=\frac{1}{2}{\left\{X^{\left(e\right)}\right\}}^{T}\left[K_{i}^{e}\right]\left\{X^{\left(e\right)}\right\}$$

(A6) $$E_{\beta 2}^{\left(e\right)}=\int_{0}^{2a}\int_{0}^{2b}\int_{\frac{-h_{2}}{2}}^{\frac{h_{2}}{2}}\left[G\beta_{x}^{2}+G\beta_{y}^{2}\right]dxdydz=\frac{1}{2}{\left\{X^{\left(e\right)}\right\}}^{T}\left[K_{\beta 2}^{e}\right]\left\{X^{\left(e\right)}\right\}$$

(A7) $$k_{i}^{\left(e\right)}=h_{i}\int_{0}^{2a}\int_{0}^{2b}B_{i}^{T}D_{i}B_{i}dxdy+\frac{h_{i}}{12}\int_{0}^{2a}\int_{0}^{2b}B^{T}D_{i}Bdxdy,k_{\beta 2}^{\left(e\right)}=Gh_{2}\int_{0}^{2a}\int_{0}^{2b}N_{10}^{T}N_{10}+N_{11}^{T}N_{11}dxdy$$

(A8) $$\left[\begin{array}{c} \sigma_{ix}\\ \sigma_{iy}\\ \tau_{ixy} \end{array}\right]=D_{i}\left[\begin{array}{c} \varepsilon_{ix}\\ \varepsilon_{iy}\\ \gamma_{ixy} \end{array}\right],\varepsilon_{ix}=\frac{\partial u_{i}}{\partial x}-z\frac{\partial^{2}w}{\partial x^{2}},\varepsilon_{iy}=\frac{\partial v_{i}}{\partial y}-z\frac{\partial^{2}w}{\partial y^{2}},\gamma_{ixy}=\frac{\partial u_{i}}{\partial y}-z\frac{\partial^{2}w}{\partial x\partial y}+\frac{\partial v_{i}}{\partial x}-z\frac{\partial^{2}w}{\partial x\partial y}$$

where I =1, 2, and 3, which, respectively, represent the piezoelectric layer, viscoelastic layer, and base layer.

$\sigma_{x},\;\sigma_{y},\;\tau_{\mathrm{xy}}$ represents normal stress in x direction, normal stress in y direction, and in-plane shear stress. $\varepsilon_{x},\;\varepsilon_{y},\;\gamma_{\mathrm{xy}}$ represents the normal strain in the x direction, the normal strain in the y direction, and the in-plane shear strain.

$$B=[N_{5},xx;N_{5}yy;2N_{5},xy],B_{1}=[N_{1},x;N_{2},y;N_{2},x+N_{1},y],B_{2}=[N_{8},x;N_{9},y;N_{8},x+N_{9},y],$$

$$B_{3}=[N_{3},x;N_{4},y;N_{3},x+N_{4},y],D_{i}=\frac{E_{i}}{1-\mu_{i}^{2}}\left[\begin{array}{ccc} 1 & \mu_{i} & 0\\ \mu_{i} & 1 & 0\\ 0 & 0 & \frac{1-\mu_{i}}{2} \end{array}\right],$$

in $B_{i}$ represent derivation.

where $E_{i}$ is Young’s modulus of each layer, $\mu_{i}$ is Poisson’s ratio of each layer,$h_{i}$ is the height of each floor, and $p_{i}$ is the density of each layer.

(A9) $$W_{c}^{\left(e\right)}=\frac{1}{2}{∭}_{v}\left(\sigma_{1x}^{E}\varepsilon_{1x}+\sigma_{1y}^{E}\varepsilon_{1y}+\tau_{1xy}^{E}\gamma_{1xy}\right)dv={\left\{X^{\left(e\right)}\right\}}^{T}F_{c}^{\left(e\right)},\;\sigma_{1}^{E}=\left[\begin{array}{c} \sigma_{1x}^{E}\\ \sigma_{1y}^{E}\\ \tau_{1xy}^{E} \end{array}\right]=D_{1}\left[\begin{array}{c} d_{11}\\ d_{22}\\ 0 \end{array}\right]\frac{V_{1}(t)}{h_{1}}$$

(A10) $$F_{c}^{\left(e\right)}=\frac{1}{2}V_{1}(t)\int_{0}^{2a}\int_{0}^{2b}B_{1}^{T}D_{1}{\left[\begin{array}{ccc} d_{11} & d_{22} & 0 \end{array}\right]}^{T}dxdy+\frac{1}{4}h_{1}V_{1}(t)\int_{0}^{2a}\int_{0}^{2b}B^{T}D_{1}{\left[\begin{array}{ccc} d_{11} & d_{22} & 0 \end{array}\right]}^{T}dxdy$$

where $V_{c}^{e}$ is the voltage value applied by the piezoelectric layer along the thickness direction and $d_{11},d_{22}$ is the piezoelectric constant.
